# The Victoria Hospital for Children

**Published:** 1899-01-07

**Authors:** 


### EDITOR'S LETTER-BOX.

[Our Correspondents are reminded that prolixity is a great bar to publication, and that brevity of style and conciseness of statement greatly facilitate early insertion.]

### THE VICTORIA HOSPITAL FOR CHILDREN.

MR. A. CAMERON SKINNER, Secretary of the Victoria Hospital, Chelsea, writes: I must call your attention to an error in your report of the meeting held on behalf of this hospital at Grosvenor House. The report states that the new building will be erected on a site at Kennington Oval, whereas the fact is that the building is to be erected on land, our own property, adjoining the present building. The cause of the error was doubtless the reference by the Duke of Westminster to the proposed removal of the Belgrave Hospital to Kennington, but it is none the less confusing, and I shall be much obliged if you will make the correction in your next issue. It is one of the most important and valuable points in the building scheme that the land adjoins the present hospital, and that we have the freehold. I shall be also obliged if you will make public the fact that the Queen has promised a donation of £100 to the building.

